# Gut Microbial Dysbiosis May Predict Diarrhea and Fatigue in Patients Undergoing Pelvic Cancer Radiotherapy: A Pilot Study

**DOI:** 10.1371/journal.pone.0126312

**Published:** 2015-05-08

**Authors:** Aiping Wang, Zongxin Ling, Zhixiang Yang, Pawel R. Kiela, Tao Wang, Cheng Wang, Le Cao, Fang Geng, Mingqiang Shen, Xinze Ran, Yongping Su, Tianmin Cheng, Junping Wang

**Affiliations:** 1 Institute of Combined Injury, State Key Laboratory of Trauma, Burns and Combined Injury, Chongqing Engineering Research Center for Nanomedicine, College of Preventive Medicine, Third Military Medical University, Chongqing, China; 2 State Key Laboratory for Diagnosis and Treatment of Infectious Diseases, First Affiliated Hospital, School of Medicine, Zhejiang University, Hangzhou, China; 3 Department of Oncology, Chongqing Zhongshan Hospital, Chongqing, China; 4 Departments of Pediatrics & Immunobiology, College of Medicine, the University of Arizona, Tucson, Arizona, United States of America; Fox Chase Cancer Center, UNITED STATES

## Abstract

Fatigue and diarrhea are the most frequent adverse effects of pelvic radiotherapy, while their etiologies are largely unknown. The aim of this study is to investigate the correlations between fatigue, diarrhea, and alterations in gut microbiota induced by pelvic radiotherapy. During the 5-week treatment of pelvic radiotherapy in 11 cancer patients, the general fatigue score significantly increased and was more prominent in the patients with diarrhea. The fatigue score was closely correlated with the decrease of serum citrulline (an indicator of the functional enterocyte mass) and the increases of systemic inflammatory proteins, including haptoglobin, orosomuoid, α_1_-antitrypsin and TNF-α. Serum level of lipopolysaccharide (LPS) was also elevated, especially in the patients with diarrhea indicating epithelial barrier breach and endotoxemia. Pyrosequencing analysis of 16S rRNA gene revealed that microbial diversity, richness, and the *Firmicutes/Bacteroidetes* ratio were significantly altered prior to radiotherapy in patients who later developed diarrhea. Pelvic radiotherapy induced further changes in fecal microbial ecology, some of which were specific to the patients with or without diarrhea. Our results indicate that gut microbial dysbiosis prior to radiation therapy may be exploited to predict development of diarrhea and to guide preventive treatment options. Radiation-induced dysbiosis may contribute to pelvic radiation disease, including mucositis, diarrhea, systemic inflammatory response, and pelvic radiotherapy-associated fatigue in cancer patients.

## Introduction

Pelvic cancers are among the most frequently diagnosed cancers worldwide[1], and pelvic radiotherapy is often an integral part of the multidisciplinary approaches used to treat pelvic tumors [2,3]. However, adverse effects following pelvic radiotherapy represent major complications and have been collectively termed pelvic radiation disease (PRD) [4]. Fatigue is a prevalent PRD symptom, which affects patients’ physical and psychosocial well-being, consequently resulting in a cluster of other symptoms, such as pain, sleeplessness, and anxiety [5]. Radiation enteropathy (mucositis), frequently accompanied by diarrhea, is another PRD symptom [6], which further impairs the quality of life [7]. Moreover, fatigue and enteropathy may represent interdependent symptoms as suggested by the prospective study by Jakobsson et al. [8] with women undergoing pelvic radiotherapy for anal or uterine cancer.

Considerable progress towards reducing toxicity of radiation therapy has been made in recent years, especially by increasing the precision of delivery of the radiation beam, and by pharmacological interventions [6]. However, our incomplete understanding of the precise etiology and inter-relatedness within the complex network of pelvic radiation disease symptoms hinders the development of optimized prevention strategies [9].

Recent explosion in knowledge stemming from culture-independent analysis of gut microbiota clearly points to the critical role of balanced mutualistic microbe-host interactions as an integrative point in the pathogenesis of not just the local intestinal inflammatory tone, but also in regulating systemic metabolism, extraintestinal inflammatory diseases, as well as the function of the central nervous system [10]. The gut microbiota is a dynamic ecosystem and is highly susceptible to environmental influences such as dietary factors or inflammation. In a small study, Manichanh et al. [11] showed that patients which suffered acute post-radiotherapy diarrhea had altered gut microbial diversity, an observation recently recapitulated in mice [12]. Germ-free mice were more resistant to radiation-induced enteritis [13], whereas manipulation of gut microbiota through administration of probiotics improved the gastrointestinal toxicity of radiotherapy [14,15]. These published data, along with the emerging relevance of gut microbiota in the pathogenesis of Inflammatory Bowel Diseases (IBD) suggests that gut microbial ecology may represent not only a potential tool for risk assessment, but also for reducing the symptoms of PRD via manipulation of the intestinal flora [16].

Interestingly, inflammatory response caused by the release of inflammatory mediators has been proposed to contribute to the development of fatigue [9]. Schubert et al. [17] described a significant correlation between fatigue and the release of IL-6. Moreover, intensity of fatigue is positively correlated with the severity of diarrhea [5]. Collectively, the available evidence suggests that fatigue, mucosal injury and diarrhea, and dysbiosis are really a continuum rather than a set of independent symptoms. However, no comprehensive and integrative molecular analyses have been performed to investigate gut microbial ecology along with fatigue and diarrhea induced by pelvic radiotherapy. In this study, we provide the first attempt to link those PRD symptoms through 16S rRNA-based microbial profiling, and describe associations among gut microbial dysbiosis and fatigue and diarrhea. Our pilot study also suggests that pre-existing changes in microbial gut ecology in cancer patients may not only play an etiological role in the development of post-radiation enteropathy, but that they may serve as a predictive tool, which may guide clinical approaches to the PRD prevention.

## Materials and Methods

### Patient demographics and sampling

A total of 20 patients, scheduled to receive pelvic radiotherapy for the first time at the Chongqing Zhongshan Hospital (China), were registered for this study. Exclusion criteria included prior chemotherapy, or administration of steroids, immunosuppressants and/or antibiotics within one month prior to sample collection. Two sequential stool samples were collected from each patient at time points before and just after radiotherapy. Three blood samples were acquired from each patient at time points: immediately before, at the 3^rd^-week and 5^th^-week of radiotherapy, respectively. Additionally, 4 gender- and age- matched healthy volunteers were recruited to provide samples as controls. All samples were aliquoted and stored at -80°C until further use. This study was approved by the Ethics Committees of Chongqing Zhongshan Hospital and Third Military Medical University, and in accordance with the Declaration of Helsinki. Written informed-consent documents were obtained from all the participants in this study.

### Assessment of diarrhea and fatigue during radiotherapy

In parallel with the time points in the serum samples, each patient completed a questionnaire to establish the grade of diarrhea associated with the radiotherapy. According to the Common Terminology Criteria for Adverse Events (CTCAE), diarrhea was classified into five grades depending on the severity of patient’s condition [18]. Grade 0 indicates no diarrhea, grade 1 refers to an increase of four or fewer stools per day, grade 2 refers to an increase of four to six stools per day, and grade 3 refers to an increase of seven or more stools per day. Grade 4 indicates life-threatening consequences.

The intensity of general fatigue in this study was assessed using the modified Multidimensional Fatigue Inventory (MFI-20) subscale [19], which consists of four statements (I feel fit, I feel tired, I am rested, and I tire easily), The statements are rated against a five-point scale from “yes, that is true” to “no, that is not true”, and reported over time. The score ranges from 4 to 20 is based on patients’ reports of fatigue in face-to-face interviews, whereby a higher score indicates greater fatigue.

### Biochemical marker measurement

Serum citrulline, orosomucoid, haptoglobin and α_1_-antitrypsin were determined using ELISA kits (USCN Life Science and Technology Co. Ltd, Wuhan, China). Serum lipopolysaccharide (LPS) was also measured using an ELISA kit (R&D System, Inc., Minneaplis, MN). TNF-α was measured using a microfluidic chip kit (Millipore, Inc., Billerica, MA). The intra- and inter-assay coefficients of variation were <5% and <10%, respectively. Serum samples were diluted appropriately and assayed according to the manufacturer’s instructions.

### Pyrosequencing and bioinformatics analysis

The stool samples from the patients and 4 healthy volunteers were collected at designated time points. Bacterial genomic DNA was extracted from stool samples using a DNA Mini Kit (QIAGEN, Hilden, Germany) according to the modified manufacturer’s instructions [20]. Universal bacterial primers, which correspond to positions 341–534 of the conserved *Escherichia coli* 16S rRNA gene sequence, were used to amplify the 16S rRNA gene hypervariable V3-region. PCR amplicon libraries was created for each individual DNA sample. Prior to sequencing, each PCR product was purified with the Gel Extraction Kit (QIAGEN, Hilden, Germany) and quantified using a NanoDrop ND-1000 spectrophotometer (Thermo Electron Corporation). Amplicon pyrosequencing was performed with standard 454/Roche GS-FLX Titanium protocols, where equimolar amounts of PCR amplicon libraries from each sample were pooled. A set of 8-bp barcodes designed by Fierer et al. [21] was used to pool and sort multiple samples in a single 454 GS-FLX run.

High-quality pyrosequencing reads were screened out according to barcode- and primer-sequence filtering as previously described [22]. Sequences were aligned in with SILVA ribosomal RNA database and clustered into operational taxonomic units (OTUs) [23]. The OTUs that reached at a 97% similarity level were used for alpha diversity (Shannon), richness (Chao), Good’s coverage, and rarefaction curve analysis using the mothur software [24]. Taxonomy-based analyses were performed by classifying each sequence using the Naïve Bayesian Classifier program of the Michigan State University Center for Microbial Ecology Ribosomal Database Project database (http://rdp.cme.msu.edu) with a 50% bootstrap score [25]. Phylogenetic beta diversity was measured using OTUs for each sample using the mother software [26].

### Statistical analysis

Statistical analysis of the non-sequencing data was performed using the SPSS Software Package (SPSS version 17.0, IBM, Armonk, NY). Results are expressed as means ± standard deviation (SD). The statistical analysis was carried out with ANOVA followed by Tukey post-hoc test, paired or unpaired *t*-test or non-parametric Wilcoxon signed-rank test, as appropriate. Correlations were calculated with the Pearson and partial correlation tests. Values of *P* ≤ 0.05 were considered statistically significant.

### Accession numbers

The sequence data from this study has been deposited in the GenBank Sequence Read Archive with the accession number SRP035279.

## Results

### Patient characteristics

Twenty patients were screened over the course of radiotherapy. Nine patients, who took steroids, immunosuppressants and/or antibiotics within one month of sample collection, or failed to supply the required questionnaires, and/or provide sequential samples, were excluded from the study. 11 patients were included in the final analysis, of whom eight females suffered from cervical cancer, one female from anal cancer and two males from colorectal cancer. Median age of the cohort was 51 years (range, 41–64 years). None of those patients received chemotherapy, and underwent their first conventional radiotherapy at a dosage of 1.8–2.0 Gy/day, five times a week during the 5-week period (a cumulative dosage of 44–50 Gy) (Table 1).

**Table 1:** Patient characteristics.

Patient	Sex	Age	Body Mass Index	Diagnosed Cancer	Dose of Pelvic Radiotherapy (Gy)	Grade of Diarrhea^▲^
P1	F	47	21.64	Cervical squamous carcinoma	48	0-0-0
P2	F	51	20.40	Cervical cancer	50	0-0-0
P3	F	50	20.50	Cervical squamous carcinoma	46	0-0-0
P4	F	64	24.03	Anal cancer	48	0-0-0
P5	F	41	20.81	Cervical squamous carcinoma	44	0-0-0
P6	M	47	21.97	Colorectal cancer	46	0-3-2
P7	M	56	22.04	Colorectal cancer	48	0-3-1
P8	F	62	20.81	Cervical cancer	44	0-2-2
P9	F	45	22.29	Cervical squamous carcinoma	50	0-3-2
P10	F	65	21.47	Cervical squamous carcinoma	46	0-2-1
P11	F	56	21.37	Cervical cancer	44	0-3-2

^▲^Grade of diarrhea was assigned based on the Common Terminology Criteria for Adverse Events and reported at the three time points: prior to, at the third, and the fifth week of radiotherapy [18]. Grade 0: no diarrhea; grade 1: increase of <4 stools/day; grade 2: increase of 4–6 stools/day or nocturnal stools; grade 3: increase of ≥ 7 stools/day or incontinence or need for parenteral support for dehydration; grade 4: physiologic consequences requiring intensive care or hemodynamic collapse.

### Occurrences of fatigue and diarrhea induced by pelvic radiation in the patients

**Fig 1:**
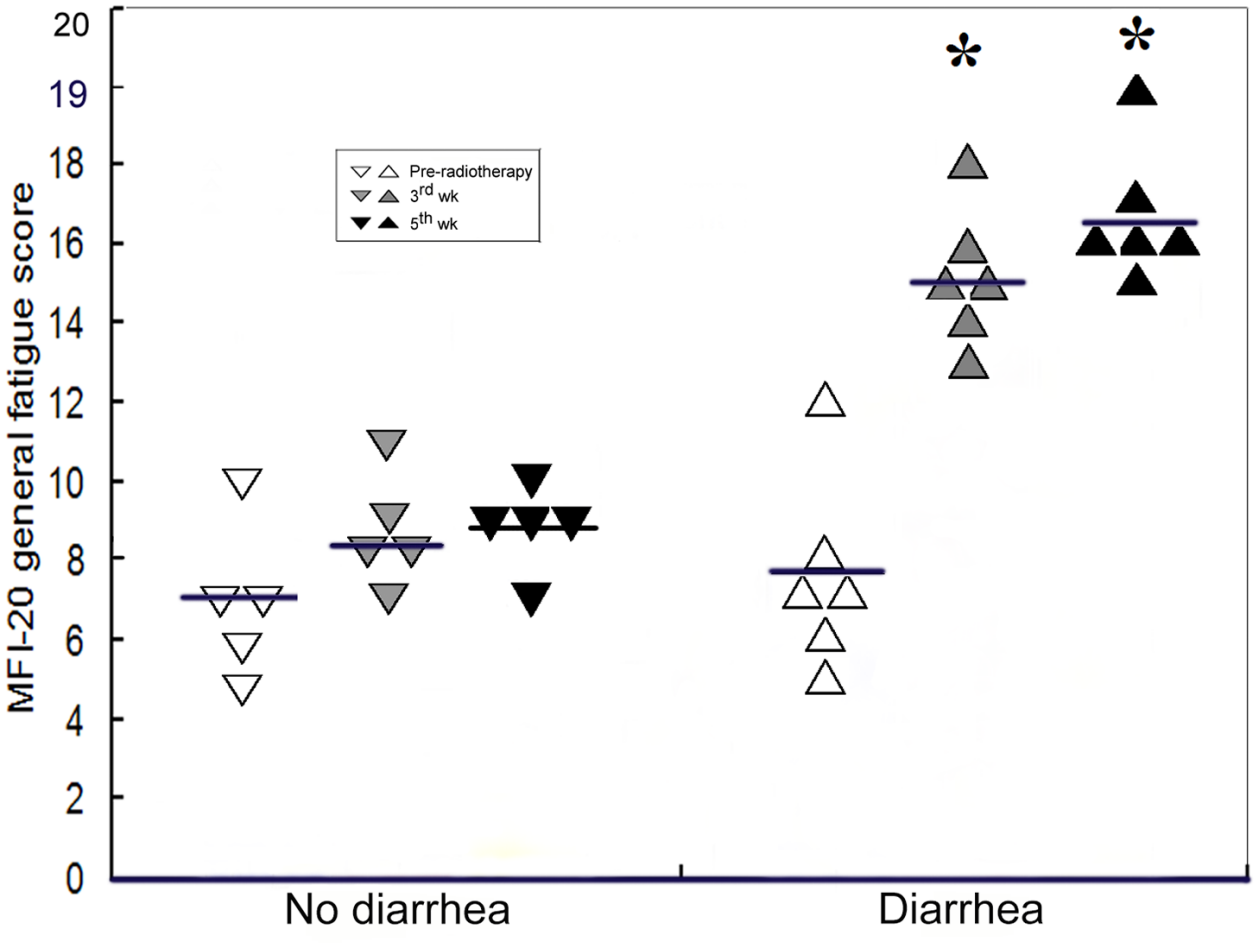
General fatigue scores in different patient groups at indicated time points. Each symbol represents a sample. Horizontal bar lines refer to mean value in each group. * indicates statistical differences between pre-treatment time point and 3^rd^ or 5^th^ week, respectively, in patients who developed diarrhea (p < 0.01, n = 6; paired *t*-test).

The summary of the questionnaires concerning adverse effects of pelvic radiotherapy reflected a common symptom of fatigue among all subjects. According to the criteria of CTCAE diarrhea grade, 6 out of 11 patients suffered diarrhea with varying severity recorded on the 3^rd^ and 5^th^ week of radiotherapy (Table 1). Prior to treatment, the MFI-20 general fatigue score was identical in patients who did or did not develop symptoms of diarrhea post-radiotherapy (Fig 1). In patients without diarrhea, the fatigue score increased slightly after radiotherapy, but it did not reach statistical significance (Fig 1). However, patients with diarrhea reported dramatically increased fatigue score at both third and fifth week of radiotherapy (*p*<0.01; Fig 1), thus suggesting that diarrhea contributes to the patients’ fatigue during radiotherapy.

### Changes in serum levels of biomarkers in response to pelvic radiation therapy and their correlation with fatigue and diarrhea

**Fig 2:**
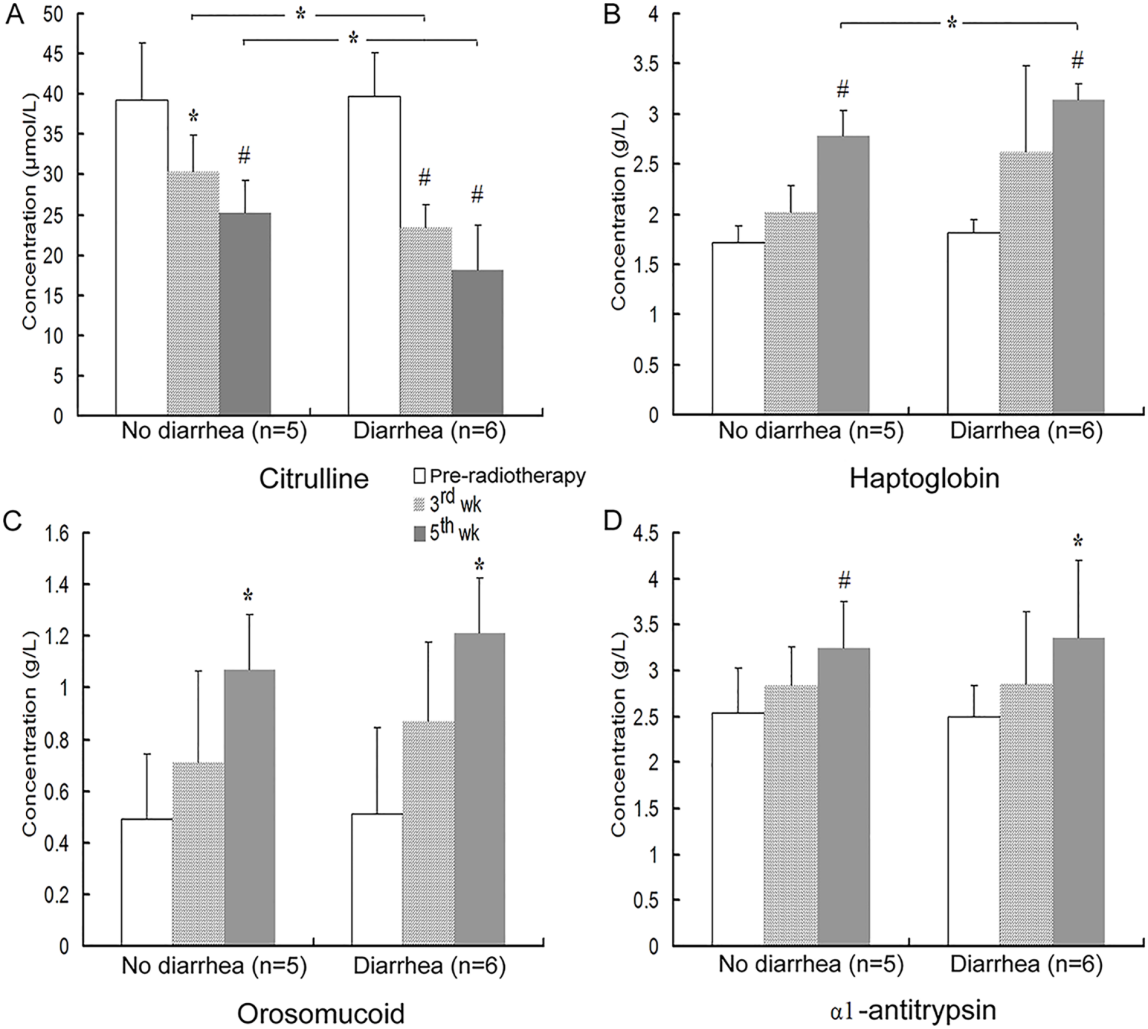
Biochemical systemic markers of enterocyte mass and inflammation in different patient groups at indicated time points. Serum citruline, haptoglobin, orosomucid, and α1-antitrypsin were analyzed by ELISA, as described in Materials and Methods section. Differences within groups were analyzed by ANOVA followed Tukey post-hoc test. Differences at respective time points between the two groups were analyzed with were analyzed with unpaired *t*-test, *p<0.05, ^#^ *p*<0.01.

To explore the etiopathogenesis of radiation-induced fatigue and diarrhea, serum levels of biomarkers related to intestinal injury and systemic inflammation were analyzed. Plasma citrulline, a nitrogen end product of glutamine metabolism in the enterocytes, is an established indicator of the functional enterocyte mass [27], and has been described as a surrogate marker of radiation-induced small bowel mucosal atrophy [28]. Serum citrulline concentration significantly decreased during radiotherapy in all patients regardless of the ensuing diarrhea (Fig 2A). However, it was significantly lower in patients with diarrhea both three and five weeks into radiotherapy (Fig 2A). These results suggest that diarrhea was associated with a more significant intestinal epithelial atrophy during pelvic radiotherapy. Moreover, we observed a significant negative correlation (*r* = -0.648, *p* = 0.031; *r* = -0.658, *p* = 0.028 for 3^rd^ and 5^th^ wk respectively) between MFI-20 general fatigue scores and citrulline concentration in the following 5-week treatment.

All three acute phase proteins selected as systemic markers of inflammation, haptoglobin, orosomucoid and α_1_-antitrypsin, were significantly and time-dependently elevated in all patients receiving pelvic radiotherapy, reaching statistical significance at the 5^th^ week time point (p<0.05; Fig 2B–2D). Of the three proteins, only haptoglobin showed significant further increase in patients who developed diarrhea during 3- to 5- week treatment (Fig 2B). At the 5^th^ week time point, general fatigue was positively correlated with haptoglobin (*r* = 0.796, *p* = 0.003), with strong partial correlation under control variables of orosomucoid and α1-antitrypsin (*r* = 0.718, *p* = 0.029). Significant positive correlations were also found between general fatigue scores and concentrations of serum orosomucoid at both of the 3^rd^ and 5^th^ week time points (*r* = 0.612, *p* = 0.045 and *r* = 0.630, *p* = 0.038, respectively). These results suggest that boosting fatigue during radiotherapy could be at least partly attributed to systemic inflammation induced by pelvic radiation.

**Fig 3:**
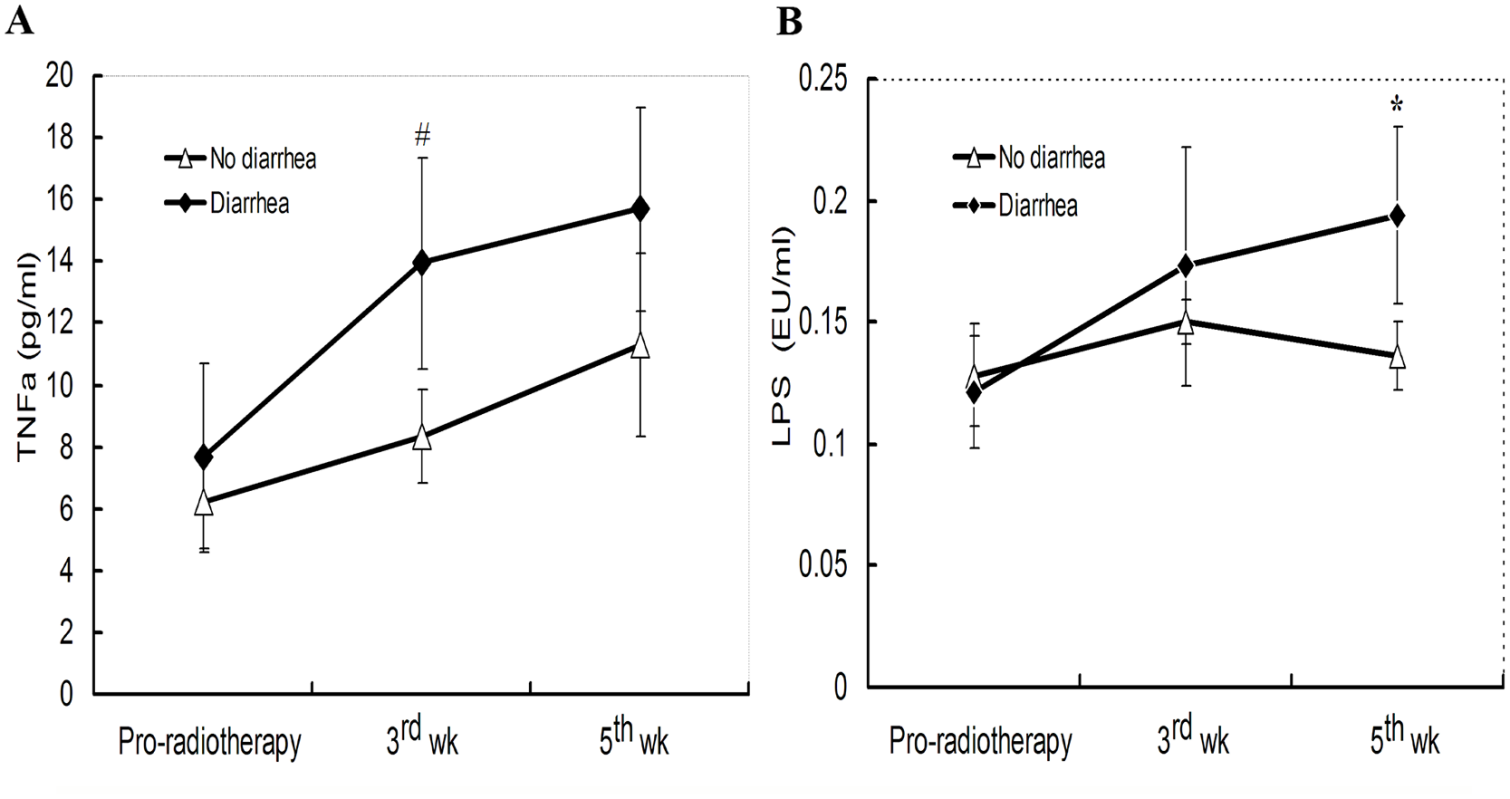
Serum concentrations of TNFα (A) and LPS (B) in patients receiving pelvic radiotherapy. TNF and LPS were analyzed by ELISA, as described in Materials and Methods section. *^, #^ indicates statistical differences between “No diarrhea” and “Diarrhea” groups at the respective time point (*p <0.05, ^#^ *p*<0.01, non-parametric paired *t*-test, n = 5–6).

Serum TNFα concentration showed an increasing trend in all patients receiving pelvic radiotherapy, with the cytokine concentration significantly higher in patients with diarrhea, especially at the 3^rd^ week (*p*<0.01) (Fig 3A). While serum LPS levels was not affected by radiotherapy in patients who did not develop diarrhea, an evidence of endotoxemia was documented in patients with diarrhea, with the statistical difference between the two groups reached at 5^th^ week treatment (*p*<0.05) (Fig 3B).

### Altered gut microbial ecology in the cancer patients prior to pelvic radiotherapy

**Fig 4:**
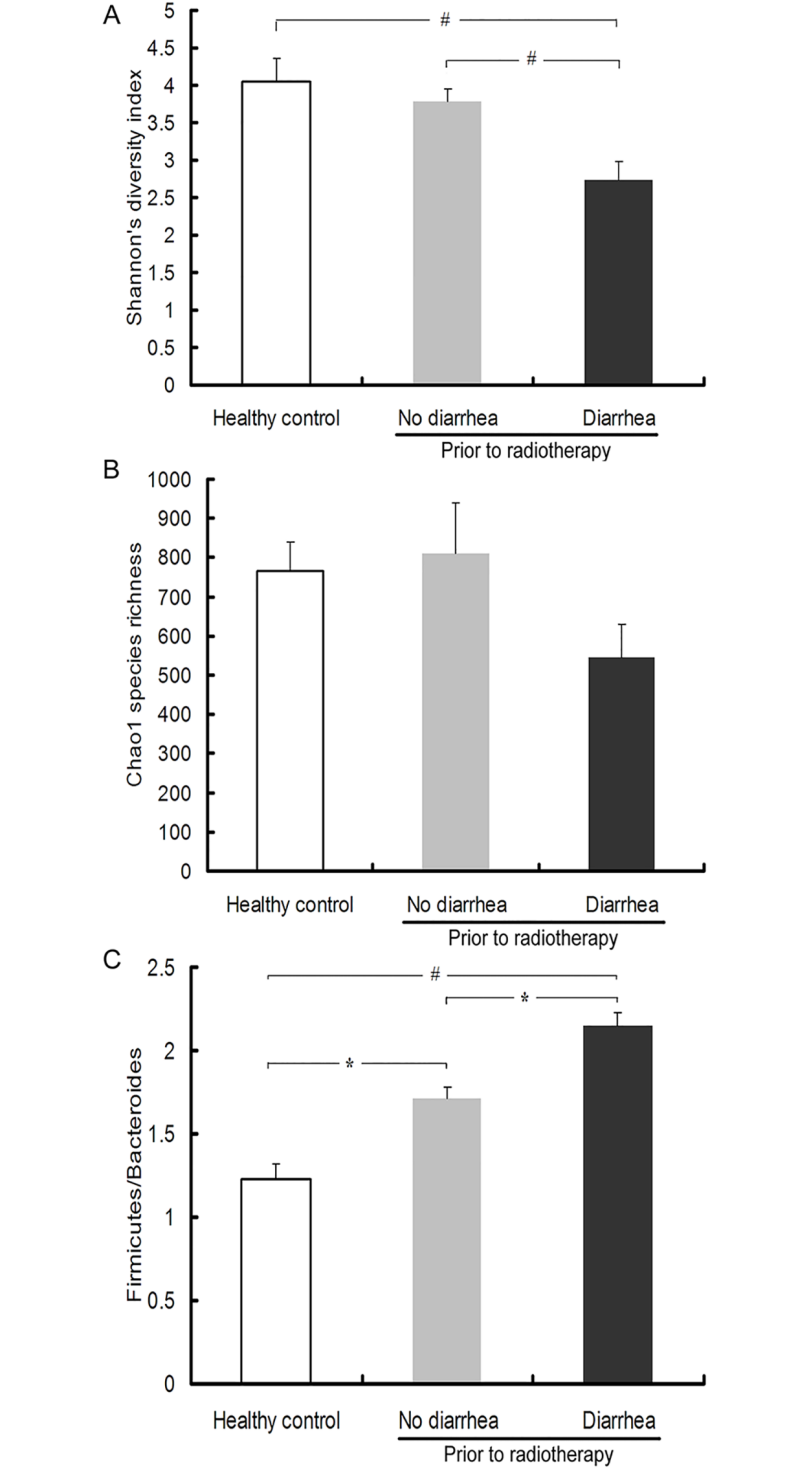
Fecal microbial ecology is altered in cancer patients prior to radiotherapy. Differences in microbial alpha diversity between healthy controls and cancer patients prior to radiotherapy who did or did not develop diarrhea is indicated by Shannon’s diversity index (A) and Chao1 species richness (B). (C) *Firmicutes/Bacteroides* ratio in the same groups of patients. * and ^#^ indicate statistically significant differences at *p*<0.05 and 0.01, respectively (ANOVA followed by Tukey post-hoc test).

We compared the stool microbiome profile between the patients and 4 healthy volunteers. A total of 170,021 high-quality sequences were obtained in this study. The Good’s coverage of each group was over 95%, showing that the 16S rRNA sequences identified in these groups represented the majority of gut bacteria appearing in the samples (Table 2). Analysis of alpha diversity showed that prior to radiotherapy, patients who later progressed to diarrhea had significantly lower fecal microbial diversity not only as compared to healthy controls (*p*<0.01), but more importantly, as compared to patients who did not develop diarrhea (*p*<0.01). This was especially pronounced in Shannon diversity index, which accounts for both abundance and evenness of the species present (Fig 4A). Similar trend was observed for Chao1 species richness index, albeit without reaching statistical significance (Fig 4B).

**Table 2:** Pyrosequencing data summary.

Group	Diarrhea	Sequences	Good (%)	OTUs	Chao1	Shannon
Healthy controls (n = 4)	No	4245±415	94.97±0.37	411±48	764.79±152.90	4.05±0.31
Pre-radiotherapy (n = 5)	No	5191±765	95.76±0.58	388±57	810.49±285.76	3.78±0.18
Post-radiotherapy (n = 5)	No	6404±1371	96.58±0.94	344±53	714.92±209.62	2.94±0.21^▲^ ^★^
Pre-radiotherapy (n = 6)	Yes	5335±601	97.21±0.46	254±31	544.69±205.27^★^	2.74±0.24^★^
Post-radiotherapy (n = 6)	Yes	5709±684	97.51±0.33	253±37	538.15±193.11^★^	1.94±0.26^▲^ ^★^ ^●^

The number of operational taxonomic units (OTUs), the coverage percentage (Good), species richness estimator Chao1, and Shannon’s diversity index were calculated using the Good’s method and the MOTHUR program at 3% dissimilarity level.

^▲^and^★^ show statistically significant difference compared with the corresponding pre-radiotherapy group and healthy group (paired- or unpaired *t*-test), respectively;

^●^ demonstrates significant difference of post-radiotherapy between “diarrhea” and “no diarrhea” groups (unpaired *t*-test). (*p*<0.05).

**Fig 5:**
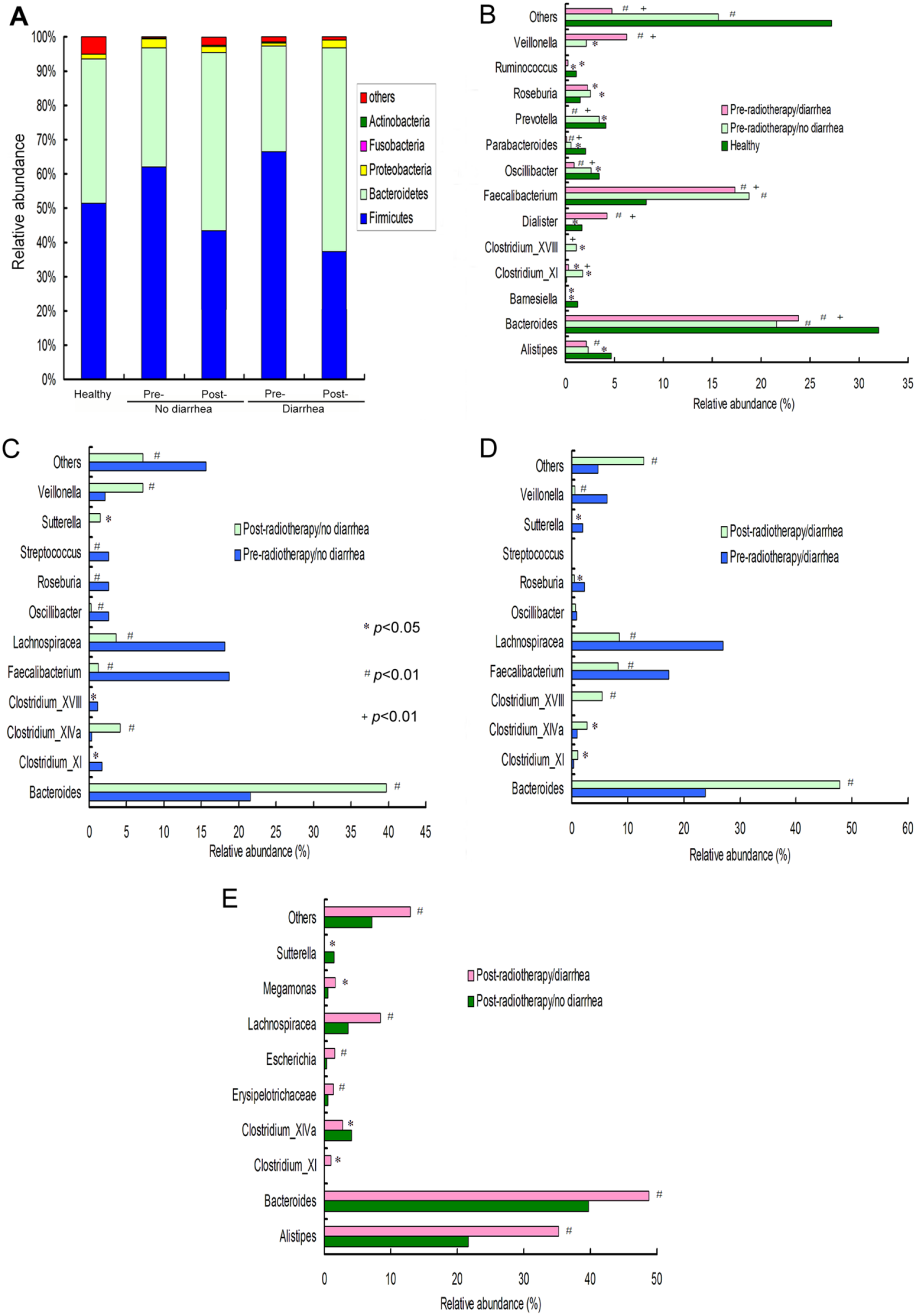
Taxonomic analysis (relative abundance) of fecal microbiota at phylum and genus levels. (**A**) relative abundance of five main bacterial phyla as well as unclassified “Others”, which include *Chloroflexi*, *Deferribacteres*, *Chlorobi*, *Acidobacteria*, *Deinococcus-Thermus*, *Planctomycetes*, *Lentisphaerae*, *Spirochaetes*, *Synergistetes*, *Tenericutes*, *Verrucomicrobia* and *Cyanobacteria*. (**B**) Selected genera statistically different between cancer patients prior to radiotherapy and healthy controls. * and # indicate significant differenence from healthy controls (unpaired Mann-Whitney test). Symbol of + indicates statistical differences between cancer patients who did or did not develop diarrhea as a result of radiotherapy (unpaired Mann-Whitney test). (**C, D**) The effects of radiotherapy on relative microbial abundance at the genus level between patients prior and after radiotherapy within “diarrhea” and “no diarrhea” groups (paired Mann-Whitney test). (**E**) Taxonomic differences at the genus level between cancer patients without and with diarrhea after radiotherapy (unpaired Mann-Whitney test). *p <0.05, ^#^ *p* <0.01, ^+^ *p*<0.01.

In taxonomic analysis, consistently with other reports, *Firmicutes* and *Bacteroidetes* were the two dominant phyla in all groups. Interestingly, the *Firmicutes/Bacteroidetes* ratio was significantly increased as compared to healthy controls (Fig 4C), which was a reflection of increased relative abundance of *Firmicutes* and a decrease in *Bacteroidetes* phylum in cancer patients prior to radiotherapy (Fig 5A). At the genus level, the relative abundances of 14 genera were found significantly different between the healthy control and cancer patients before radiotherapy (Fig 5B). Genera *Alistipes*, *Bacteroides*, *Barnesiella*, *Oscillibacter*, *Parabacteroides*, *Prevotella* and *Ruminococcus*, which account for over 1% of the total bacteria, were less abundant in the cancer patients than those in the healthy subjects. Genera *Faecalibacterium*, *Clostridium_XI*, *Roseburia* and *Veillonella* were relatively more abundant in the cancer patients. Intriguingly, prior to radiotherapy, compared to patients who did not develop diarrhea, those who did had increased abundance of *Bacteroides*, *Dialister*, *Veillonella*, and decreased abundance of *Clostridium* XI and XVIII, *Faecalibacterium*, *Oscillibacter*, *Parabacteroides*, *Prevotella* and unclassified (Genus: others) (Fig 5B).

### Microbial community structure in the gut is significantly altered after pelvic radiotherapy

In both groups of patients (those who developed diarrhea and those who did not), radiotherapy resulted in further deterioration of microbial alpha diversity, reflected particularly by the Shannon index (Table 2). At the phylum level, although *Firmicutes* and *Bacteroidetes* remained the two dominant phyla, after radiotherapy, the relative abundance ratio of *Firmicutes* to *Bacteroidetes* changed from 1.79 to 0.83 in patients without diarrhea, and from 2.15 to 0.63 in patients with diarrhea (Fig 5A). In patients who developed diarrhea, but not in those who did not, radiotherapy resulted in a significant increase in unclassified bacteria (Phylum:others) (to 2.47% vs. 0.88%, respectively).

The microbial composition was also significantly different at the genus level prior to and post-radiotherapy in both groups of cancer patients. As shown in Fig 5C and 5D, selected genera showed similar response to radiation, regardless of the development of diarrhea, The relative abundance of *Bacteroides* and *Clostridium_XIVa* were significantly increased after radiotherapy, while other dominant genera, such as *Faecalibacterium*, *Lachnospiracea*, *Oscillibacter*, *Roseburia*, and *Streptococcus*, were decreased. However, selected genera showed an opposite response between the two cancer groups. Among those, increased in patients with diarrhea and decreased in non-diarrhea group were *Clostridium* XI and XVIII, and unclassified (others), while *Veilonella* showed the reverse trend (Fig 5C and 5D).

When the two post-radiotherapy groups of cancer patients (no diarrhea vs. diarrhea) were compared, about ten bacterial genera, which constitute over 1% of the total fecal bacteria, were significantly different (Fig 5E). The relative abundance of *Alistipes*, *Bacteroides*, *Clostridium_XI*, *Erysipelotrichaceae*, *Escherichia*, *Lachnospiracea*, *Megamonas*, and unclassified (Genus: others) were significantly higher, whereas *Clostridium_XIVa* and *Sutterella* were significantly lower in the patients who developed diarrhea, in comparison with those who did not (Fig 5E). Interestingly, some genera, such as *Alistipes*, *Bacteroides* and *Roseburia*, changed inversely after pelvic radiotherapy, in comparison with healthy controls (Fig 5B–5E).

## Discussion

The concept of radiation therapy traces back to the discovery of X-rays in 1895 by Wilhelm Röntgen, and with recent technological advances in radiation, an increasing number of patients with abdominal malignancies are treated with pelvic radiotherapy in the future. However, gastrointestinal symptoms induced by pelvic radiotherapy remain a long-standing and unresolved problem. Especially, fatigue and diarrhea, which are frequently experienced by pelvic radiotherapy patients, have harmful impact on the quality of patients’ life and not infrequently lead to the suspension of treatment [29]. In this pilot study, we assessed the symptoms of fatigue and diarrhea, inflammatory markers, and fecal microbial ecology of 11 cancer patients over their five-week pelvic radiotherapy course. Within the limitation of the small patient cohort, we describe that fatigue is positively correlated with diarrhea and systemic markers of inflammation. Moreover, we showed that gut microbiota was not only affected by radiotherapy, but more importantly, that pre-existing changes in gut microbial ecology may serve as a predictive tool to identify patients who are more likely to progress to diarrhea after pelvic irradiation.

We found that the serum levels of haptoglobin, orosomucoid and α_1_-antitrypsin, the markers of systemic inflammation [30], were significantly elevated after pelvic radiotherapy. The positive correlations between fatigue and the elevation of these three proteins suggest that inflammation may contribute to the experienced fatigue. Consistent with an earlier study [5], we also observed that patients with diarrhea suffered from more severe fatigue. It is plausible that these patients experience more severe inflammation, as the serum levels of the inflammatory biomarkers were elevated more significantly in patients reporting higher fatigue scores (Figs 2 and 3). This also suggests that inflammation actuates the pathophysiology of diarrhea.

The etiology of aberrant inflammation, commonly encountered in patients during radiotherapy, is likely multifactorial. Tumor necrosis and collateral damage to normal tissues is accompanied by intestinal injury, compromised epithelial barrier and enhanced intestinal permeability, and an increased translocation of bacteria and proinflammatory luminal components. This viewpoint is supported by the findings of our study and previous reports that significant decrease in serum citrulline concentration, which indicates progressive intestinal epithelial atrophy [28,30], was accompanied by an increase in serum levels of TNFα and LPS during the treatment, especially in patients who developed diarrhea (Fig 3). The fact that endotoxemia was primarily observed in patients with diarrhea who also showed more elevated TNFα concentrations, suggests that mucositis and altered microbial-host interactions are at the heart of pelvic radiation disease.

The key role of gut microbiota in PRD has been postulated based on resistance if germ-free mice to radiation-induced enteritis [13], by post-radiotherapy changes in gut microbial diversity [11,12], and partial alleviation of the gastrointestinal toxicity of radiotherapy by probiotics [14,15]. Therefore, it was not surprising to find that radiotherapy had a profound effect on fecal microbial ecology in our patient cohort.

Several studies indicated that patients receiving cytotoxic and radiation therapy exhibit marked changes in intestinal microbiota, with most frequent decrease in *Bifidobacterium*, *Clostridium* cluster XIVa, *Faecalibacterium prausnitzii*, and increase in *Enterobacteriaceae* and *Bacteroides* (recently reviewed by Touchefeu et al. [31]). These changes may contribute to the development of mucositis, particularly diarrhea and bacteremia. In our study, a significant difference in the abundance ratio of *Firmicutes* to *Bacteroidetes* was observed in the cancer patients before and after pelvic radiotherapy. Moreover, we also found a remarkable decrease in the diversity and richness of gut flora after radiotherapy treatment, as well as significant differences in the relative abundance of selected genera between patients who developed or did not develop diarrhea. The latter finding may be related to differences in the severity of local mucosal inflammatory responses, or to changes in epithelial transport or peristalsis, factors which were not investigated in our patients.

A more striking observation is related to changes in gut microbial ecology prior to pelvic radiotherapy. Patients who progressed to diarrhea had significantly lower microbial alpha diversity and higher Firmicutes/Bacteroides ratio compared to those who did not develop diarrhea. In taxonomic analysis of the two patient groups pre-radiotherapy, we found significant differences in the relative abundance of *Veillonella*, *Prevotella*, *Parabacteroides*, *Oscillibacter*, *Faecalibacterium*, *Dialister*, *Bacteroides*, *Clostridia* clusters XI and XVIII, and unclassified (Genus: others) (Fig 5B). Although based on our results it is not possible to causatively link any of those taxa to the etiology of radiotherapy-induced diarrhea, it is of interest that *Clostridia* cluster XVIII, which has been linked to promotion of regulatory T cell expansion and protection from colitis and allergic diarrhea [32], was significantly less abundant in patients who developed diarrhea. Similar case can be made for *Faecalibacterium* genus, which includes a protective commensal, *F*. *prausnitzii*.[33] On the other hand, *Clostridium* cluster XI, which includes a known diarrheal pathogen *C*. *dificile*, although less abundant prior to radiotherapy in patients who later develop diarrhea, is significantly expanded post-therapy, while in non-diarrhea patients it followed the opposite trend. While due to the pilot nature of our study we are not able to specifically and authoritatively address the role of these changes in promoting diarrhea, inflammation, and fatigue, these results strongly suggest the need for further and more detailed studies with larger cohort of patients. Such studies, as well as studies with gnotobiotic mice with humanized microbiota, may also point to specific taxonomic changes, which may be of predictive value, and potentially guide the preventive treatment with cytoprotective, non-steroid anti-inflammatory drugs, or probiotics [34].

In summary, our study demonstrates a close association between gut microbial dysbiosis, fatigue, and diarrhea after pelvic radiotherapy, and justifies future larger scale projects in search of better predictive tools and prophylaxis of gastrointestinal radiotoxicity.
